# Supplementary material for: k-mer manifold approximation and projection for visualizing DNA sequences
Source: Genome Res. 2025 May;35(5):1234–46. doi: 10.1101/gr.279458.124 (PMC12047656; doi:10.1101/gr.279458.124)

UMAP Plot - ZBTB7C_TCGCCA40NCCT_AI_4

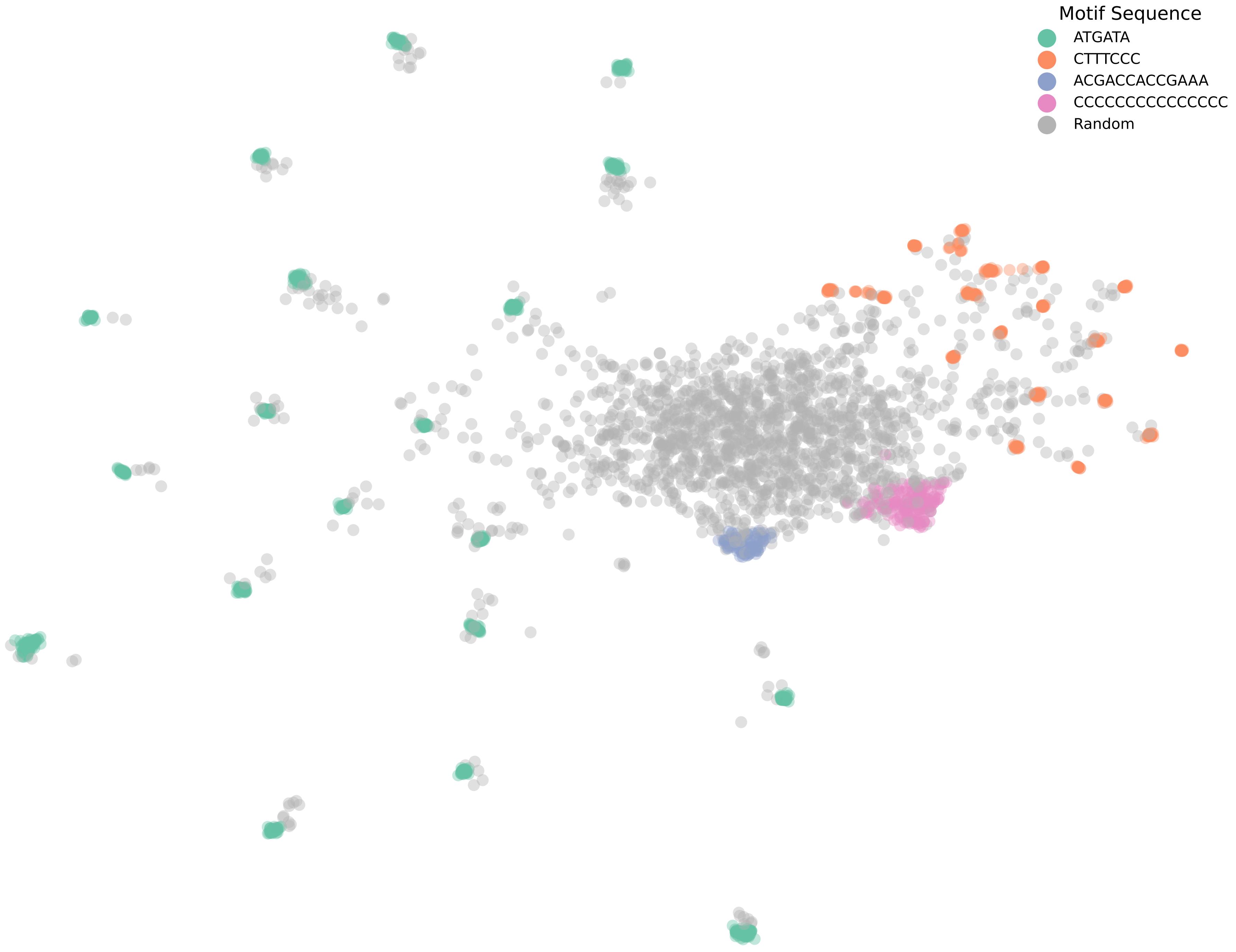

Supplement: Supplement 8 [file Supplemental_Data_1.zip › Supplemental_Data_1/ZBTB7C_TCGCCA40NCCT_AI_4/ZBTB7C_TCGCCA40NCCT_AI_4_UMAP.pdf]
